# Anti-Inflammatory Effects of Cycloheterophyllin on Dinitrochlorobenzene-Induced Atopic Dermatitis in HaCaT Cells and BALB/c Mice

**DOI:** 10.3390/molecules27092610

**Published:** 2022-04-19

**Authors:** Chia-Chen Wang, Chien-Yu Hsiao, Yu-Jou Hsu, Horng-Huey Ko, Der-Chen Chang, Chi-Feng Hung

**Affiliations:** 1 School of Medicine, Fu Jen Catholic University, New Taipei City 24205, Taiwan; jamiewang@tma.tw; 2 Department of Dermatology, Cardinal Tien Hospital, New Taipei City 23148, Taiwan; 3 Department of Nutrition and Health Science, Chang Guang University of Science and Technology, Taoyuan 33303, Taiwan; mozart@gw.cgust.edu.tw; 4 Research Center for Food and Cosmetic Safety and Research Center for Chinese Herbal Medicine, Chang Gung University of Science and Technology, Taoyuan 33303, Taiwan; 5 PhD Program in Pharmaceutical Biotechnology, Fu Jen Catholic University, New Taipei City 24205, Taiwan; s16179263@gmail.com; 6 School of Pharmacy, Kaohsiung Medical University, Kaohsiung 80708, Taiwan; hhko@kmu.edu.tw; 7 Department of Mathematics and Statistics and Department of Computer Science, Georgetown University, Washington, DC 20057, USA; chang@georgetown.edu

**Keywords:** *Artocarpus*, natural product, cycloheterophyllin, flavone, atopic dermatitis, keratinocytes, DNCB

## Abstract

Atopic dermatitis (eczema) is a condition that makes skin red and itchy. Though common in children, the condition can occur at any age. Atopic dermatitis is persistent (chronic) and tends to recur periodically. It may be accompanied by asthma or hay fever. No cure has been found for eczema. Therefore, it is very important to develop ingredients that aid the prevention and treatment of atopic dermatitis. Cycloheterophyllin is derived from *Artocarpus heterophyllus* and has antioxidant and anti-inflammatory activities. However, it still is not understood whether cycloheterophyllin is an anti-atopic dermatitis agent. Keratinocytes (HaCaT cells) and BALB/c mice for inducing AD-like cutaneous lesions were used to evaluate the potential of cycloheterophyllin as an anti-atopic dermatitis agent. The release of pro-inflammatory cytokines induced by treatment of TNF-α/IFN-γ was reduced after pretreatment with cycloheterophyllin. The inhibitory effects could be a contribution from the effect of the MAP kinases pathway. Moreover, the symptoms of atopic dermatitis (such as red skin and itching) were attenuated by pretreatment with cycloheterophyllin. Epidermal hyperplasia and mast cell infiltration were decreased in the histological section. Finally, damage to the skin barrier was also found to recover through assessment of transepidermal water loss. Taken together, prenylflavone-cycloheterophyllin from *Artocarpus heterophyllus* is a potential anti-atopic dermatitis ingredient that can be used in preventing or treating the condition.

## 1. Introduction

Nearly 10% of people have been troubled by atopic dermatitis. Lubricants and steroids are among the first-line approaches for relieving the troubles caused by atopic dermatitis [1]. In severe cases, phototherapy can be used. These disturbing symptoms are often not eradicated by these treatments. Further, in many studies, new drugs such as PD4 inhibitors or those that block IL-4 and IL-13 receptors have been shown to be very effective [2]. However, the prices of such drugs are high. They are not affordable to the general public. Because of this factor, most people can only cope with symptoms for a short period of time. In disease treatments, people seek long-term benefits in addition to short-term relief of disease symptoms. Many traditional herbal components are ingredients in medicines that treat diseases. Despite their beneficial effects, scientific evidence for their mechanisms of action and effectiveness is often lacking. Therefore, it is necessary to fill such gaps in knowledge of medicinal plants during the drug development process [3].

A variety of tropical fruit trees growing all year round produce edible fruits used by local communities in their traditional medicine. Such fruits are rich in nutrients, but their medicinal properties remain to be studied. Therefore, research on the phytochemicals of these fruit trees is necessary to promote their medical use. *Artocarpus heterophyllus* and *Artocarpus altilis* are tropical fruit trees, both of which are *Artocarpus* plants and produce edible fruits rich in protein and fiber [4] that are also very good superfood candidates [5]. In addition, the flower, heartwood, and leaves of these trees have also been found to be rich in many biologically active ingredients [4,6,7]. Among them, cycloheterophyllin is one of the ingredients with very good anti-inflammatory and antioxidant effects [8]. In past research, it was found that it has a good anti-ultraviolet damage effect and has a whitening effect by inhibiting the action of tyrosinase [8,9]. In addition, nerve research has also found that cycloheterophyllin inhibits the release of glutamate and induces anti-epileptic activity [10]. Therefore, we speculate that its anti-inflammatory and anti-allergic effects should have considerable antagonistic activity in other skin diseases, such as atopic dermatitis and psoriasis. Therefore, in this study, we preliminarily verified cycloheterophyllin’s future development potential in atopic dermatitis of the skin. We demonstrate its anti-inflammatory and anti-allergic effects using skin keratinocytes in vitro. Further, the use of DNCB-induced skin inflammation was used to test its anti-atopic dermatitis symptoms. The results show that cycloheterophyllin has good potential for future application in inflammatory skin diseases.

## 2. Results

### 2.1. The Effect of Cycloheterophyllin on Skin Cell Viability

**Figure 1:**
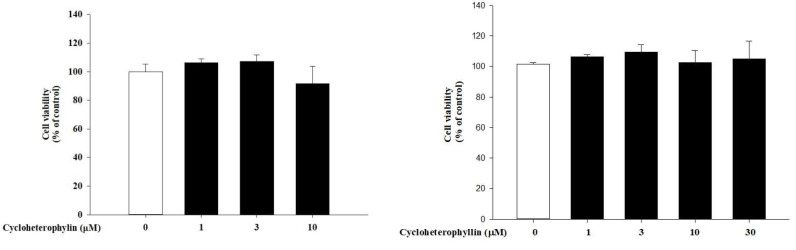
The effect of cycloheterophyllin on HaCaT cell viability. HaCaT cells were given different concentrations (1–30 μM) of cycloheterophyllin for 1 h or 24 h. The **left** panel shows the results for 1 h. The **right** panel shows the results for 24 h.

First, we examined whether cycloheterophyllin has any effect on skin keratinocyte (HaCaT cell) viability. In past studies, it has been shown that cycloheterophyllin has some anti-photoaging activity on skin fibroblasts without any toxicity [8]. In order to further understand its role in keratinocytes, we first treated HaCaT cells with cychohetrophyllin from 1 to 30 μM for 1 h or 24 h. We found that cycloheterophyllin had no significant effect on the cell viability of HaCaT cells at concentrations of 1–30 μM (Figure 1). Therefore, the following experiments related to pharmacological mechanisms were mainly conducted at concentrations of 1, 3, and 10 μM.

### 2.2. The Anti-Inflammatory Potential of Cycloheterophyllin in Tumor Necrosis Factor-α (TNF-α)/Interferon-γ (IFN-γ)-Induced Inflammatory Response in HaCaT Cells

**Figure 2:**
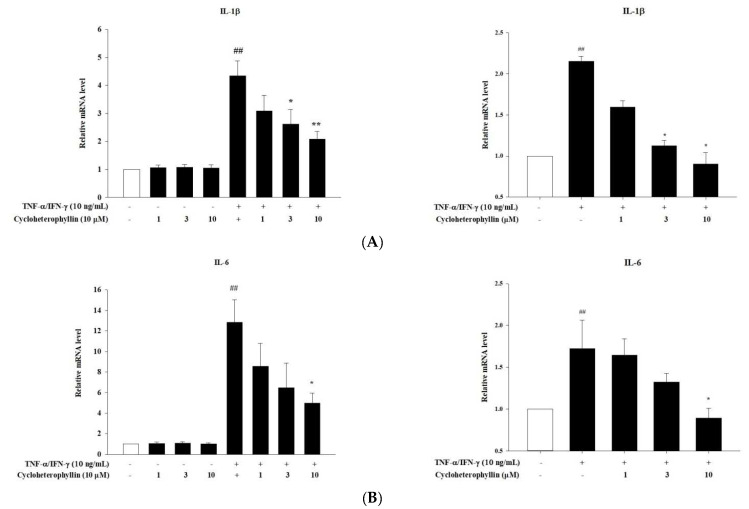

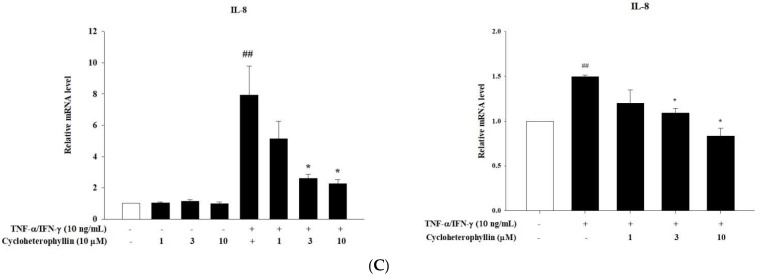
The anti-inflammatory potential of cycloheterophyllin in the TNF-α/IFN-γ-induced inflammatory response in HaCaT cells (left panel) and human epidermal keratinocytes (right panel). HaCaT cells were pretreated with different concentrations of cycloheterophyllin (1, 3, and 10 μM) for 1 h, then cells were treated with TNF-α/IFN-γ (10 ng/mL) for 1 h. Total RNA was isolated and mRNA expression levels of (**A**) IL-1β, (**B**) IL-6, and (**C**) IL-8 were determined using qPCR. Values represent mean ± SEM from three independent experiments. ^##^ *p* < 0.01 compared to untreated conditions; * *p* < 0.05 and ** *p* < 0.01 compared to TNF-α/IFN-γ treated conditions.

Upregulation of proinflammatory cytokines plays a key role in the etiology of atopic dermatitis [11]. Several studies have shown that cytokines such as interferon-γ (IFN-γ) and tumor necrosis factor-α (TNF-α) stimulate epidermal keratinocytes to activate signaling pathways involved in pro-inflammatory responses. Therefore, this model is often used as an in vitro testing method for anti-inflammatory skin treatments [12]. Stimulation of keratinocytes with TNF-α and IFN-γ results in the mRNA expression of pro-inflammatory cytokines such as IL-6, IL-1, and IL-8 (Figure 2). Cycloheterophyllin pretreatment (1, 3, and 10 μM) for 1 h significantly (*p* < 0.05) diminished the TNF-α/IFN-γ-induced mRNA expression of IL-1β, IL-6, and IL-8 (Figure 1, left panel). These results clearly demonstrate that exposure of HaCaT cells to cycloheterophyllin has a significant protective effect against inflammatory cytokines. We further performed similar experiments using primary cultured keratinocytes to confirm that this effect can also be produced in primary human skin keratinocytes. The results show that cycloheterophyllin does have the same effect on primary human keratinocytes (Figure 2, right panel).

### 2.3. The Effect of Cycloheterophyllin on the TNF-α/IFN-γ-Induced Activation of MAP Kinases in HaCaT Cells

**Figure 3:**
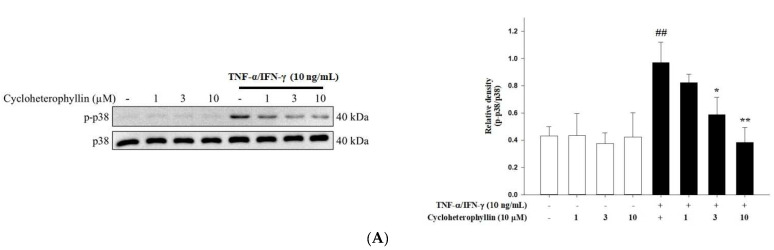

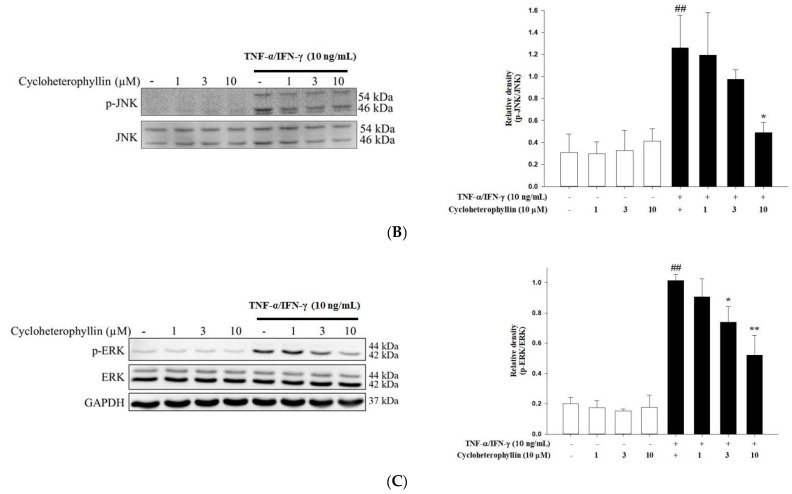
The effect of cycloheterophyllin on the TNF-α/IFN-γ-induced activation of MAP kinases in HaCaT cells. Cycloheterophyllin reduces TNF-α/IFN-γ-induced activation of p38 (**A**), JNK (**B**), and ERK (**C**) in human keratinocyte (HaCaT) cells. HaCaT cells were pretreated with various concentrations of cycloheterophyllin (1, 3, and 10 μM) for 1 h, and then cells were treated with TNF-α/IFN-γ (10 ng/mL) for 30 min. Quantitative analysis of Western blots was performed. Values represent mean ± SEM from three independent experiments. ^##^ *p* < 0.01 compared to untreated conditions; * *p* < 0.05 and ** *p* < 0.01 compared to TNF-α/IFN-γ treated conditions.

**Figure 4:**
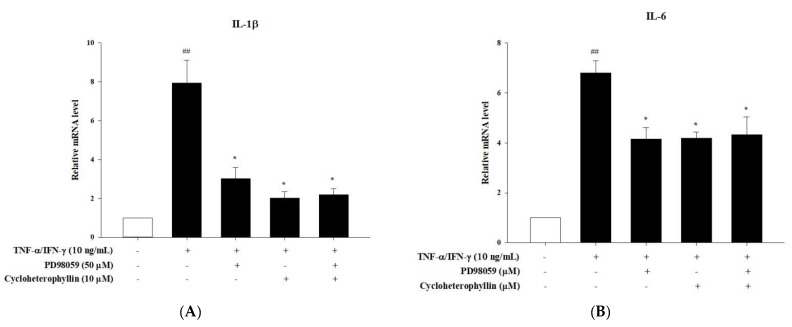

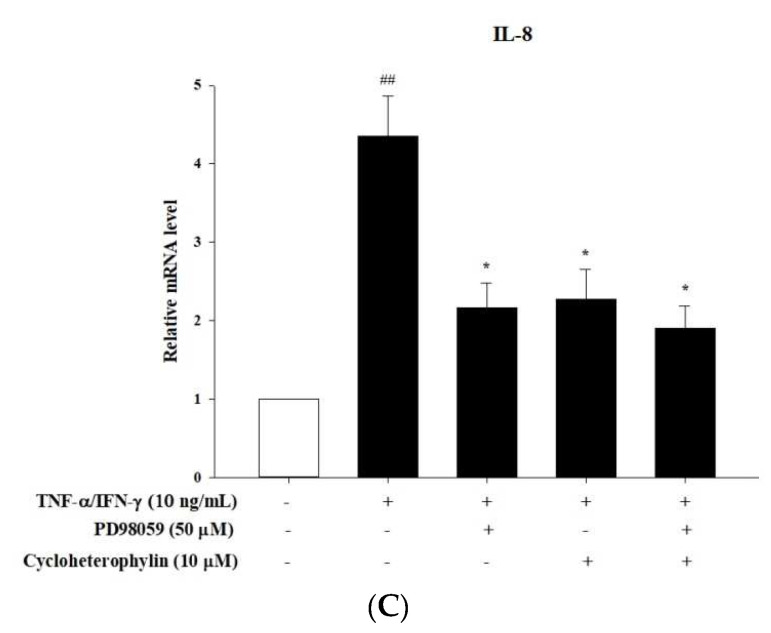
The anti-inflammatory effects of cycloheterophyllin on the TNF-α/IFN-γ-induced inflammatory response with/without pretreatment of MAPK inhibitor, PD98059, in HaCaT cells. HaCaT cells were pretreated with PD98059 (50 μM), cycloheterophyllin (10 μM), or their combinations for 1 h, and then cells were treated with TNF-α/IFN-γ (10 ng/mL) for 30 min. Total RNA was isolated and mRNA expression levels of (**A**) IL-1β, (**B**) IL-6, and (**C**) IL-8 were determined using qPCR. Values represent mean ± SEM from three independent experiments. ^##^ *p* < 0.01 compared to untreated conditions; * *p* < 0.05 compared to TNF-α/IFN-γ treated conditions.

A major pathway of MAP kinase phosphorylation is the activation of inflammatory transcription factors that act as downstream substrates of kinases to promote the secretion of inflammatory cytokines such as IL-6, IL-8, and IL-1β [12,13]. Therefore, we did find that TNF-α/IFN-γ (10 ng/mL) significantly increased the phosphorylation of P38 ERK and JNK1/2 (*p* < 0.01). The pretreatment of cycloheterophyllin (1, 3, and 10 μM) decreased the TNF-α/IFN-γ-induced phosphorylation of P38, ERK, and JNK in a dose-dependent manner (Figure 3). Furthermore, after pretreatment with the MAPK inhibitor, PD98059, we did not find that cycloheterophyllin could further inhibit the expression of proinflammatory cytokine mRNA (Figure 4). These experiments demonstrate that cycloheterophyllin reduces proinflammatory cytokine expression by inhibiting the phosphorylation of MAPK’s signal pathway.

### 2.4. Effects of Cycloheterophyllin on Atopic Dermatitis (AD)-like Skin Lesion in BALB/c Mice

**Figure 5:**
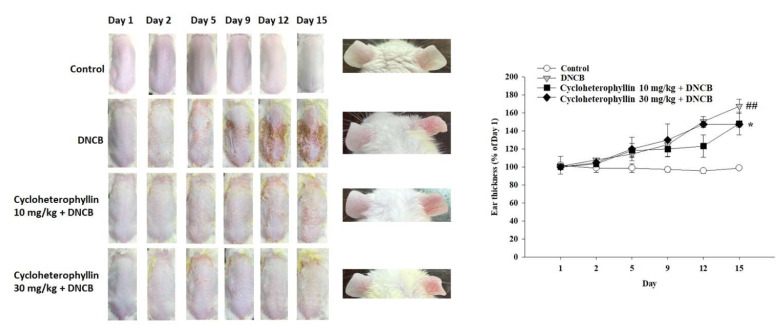
The effect of cycloheterophyllin on DNCB-induced inflammatory response to changes in skin appearance in mice. (**Left** panel) shows the effect of the skin changes on the dorsal skin. (**Middle** panel) shows the inhibitory effect of the inflammatory response on the ear. (**Right** panel) shows statistical results of ear inflammation thickness. On the first day, 1% DNCB was administered to the dorsal skin. Then, 0.5% DNCB was administered on the 8th day, the 11th day, and the 14th day to induce skin inflammation. The dorsal skin was given 100 μL DNCB and the ear was given 20 μL DNCB. ^##^ *p* < 0.01 compared to untreated conditions; * *p* < 0.05 compared to DNCB-induced group.

To investigate the anti-inflammatory effect of cycloheterophyllin on AD, a BALB/c AD model was set up by applying DNCB on the mice for fifteen days. By repeatedly exposing the dorsal skin and ear areas of mice to DNCB, AD-like skin damage was induced. During the experiment, we recorded the changes in the appearance of the skin on the backs and ears of the mice. The results show that the dorsal skin of the mice in the DNCB-induced group had serious redness, inflammation, and desquamation, while the mice in the cycloheterophyllin treatment group (10, 30 mg/kg) had reduced redness and inflammation (Figure 5, left panel). In addition, DNCB-induced ear inflammation and swelling were clearly suppressed in the cycloheterophyllin-treated group (Figure 5, middle and right panel). Observing changes in the appearance of the skin in mice, we did demonstrate that cycloheterophyllin has an anti-atopic dermatitis effect.

### 2.5. Effects of Cycloheterophyllin on Skin TEWL and Hydration in BALB/c Mice

**Figure 6:**
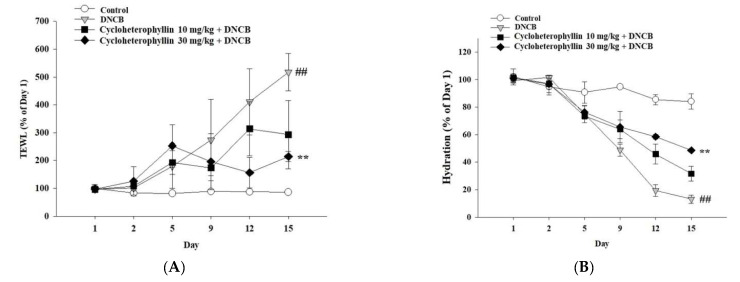
Changes in DNCB-induced skin physiological parameters of BALB/c mice after cycloheterophyllin pretreatment. Analysis of the effects of changes in transepidermal water loss (**A**) and hydration (**B**) in an atopic dermatitis-like phenotype in BALB/c mice. Values represent mean ± SEM from at least three independent experiments. ^##^ *p* < 0.01 compared to untreated conditions; ** *p* < 0.01 compared to DNCB-induced group.

Transepidermal water loss (TEWL) is the amount of water that passively evaporates to the external environment due to the water vapor pressure gradient on both sides of the skin barrier through the skin, and is used to characterize skin barrier function [14]. From the appearance of the skin of the mice that were damaged by the administration of DNCB (Figure 5), it can be seen that the damage to the skin barrier is severe: TEWL increased significantly on the fifth day. This phenomenon was continued until the DNCB was discontinued. Therefore, the skin barrier was no longer able to maintain skin moisture after the fifth day (Figure 6A). Further, we also measured that the water content of the stratum corneum reduced substantially on fifth day (Figure 6B). In the group that received cycloheterophyllin pretreatment, we did find that the reduction in TEWL and skin hydration caused by DNCB was significantly restored by day 15 (Figure 6A,B).

### 2.6. Effects of Cycloheterophyllin on DNCB-Induced Scratching Behavior and Enlarged Spleen in BALB/c Mice

**Figure 7:**
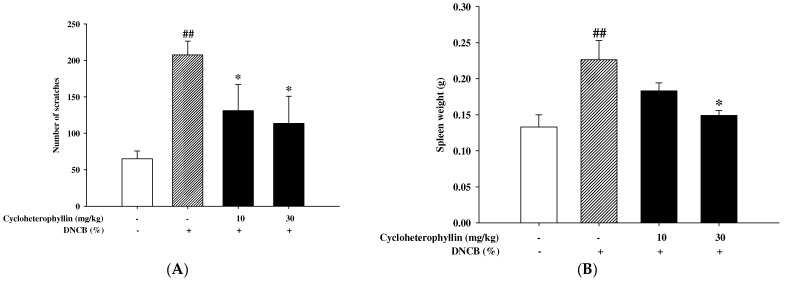
Effects of cycloheterophyllin on scratching and spleen enlargement in mice. (**A**) Analysis of changes in the number of scratches in BALB/c mice with atopic dermatitis-like appearance. (**B**) The effect of cycloheterophyllin on spleen weight. Values represent mean ± SEM from at least three independent experiments. ^##^ *p* < 0.01 vs. untreated condition; * *p* < 0.05 vs. DNCB-induced group.

Atopic dermatitis (AD) is a chronic, relapsing pruritic inflammatory skin disease. In our experiments, we also found a significant increase in the frequency of scratching in a DNCB-induced mouse model of atopic dermatitis (Figure 7A). The increased frequency of scratching was reduced by the administration of cycloheterophyllin (10 mg/kg and 30 mg/kg) (Figure 7A). Further, we also found that the weight of the spleen after treatment of DNCB was also reduced due to the pretreatment of cycloheterophyllin (Figure 7B).

### 2.7. Effects of Cycloheterophyllin on Epidermal Thickness and Mast Cell infiltration in DNCB-Induced Atopic Mice

**Figure 8:**
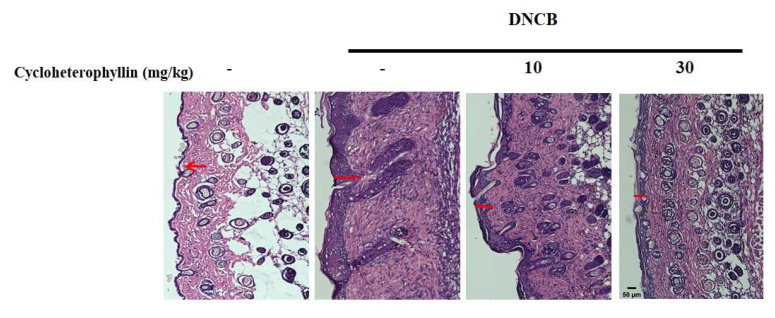

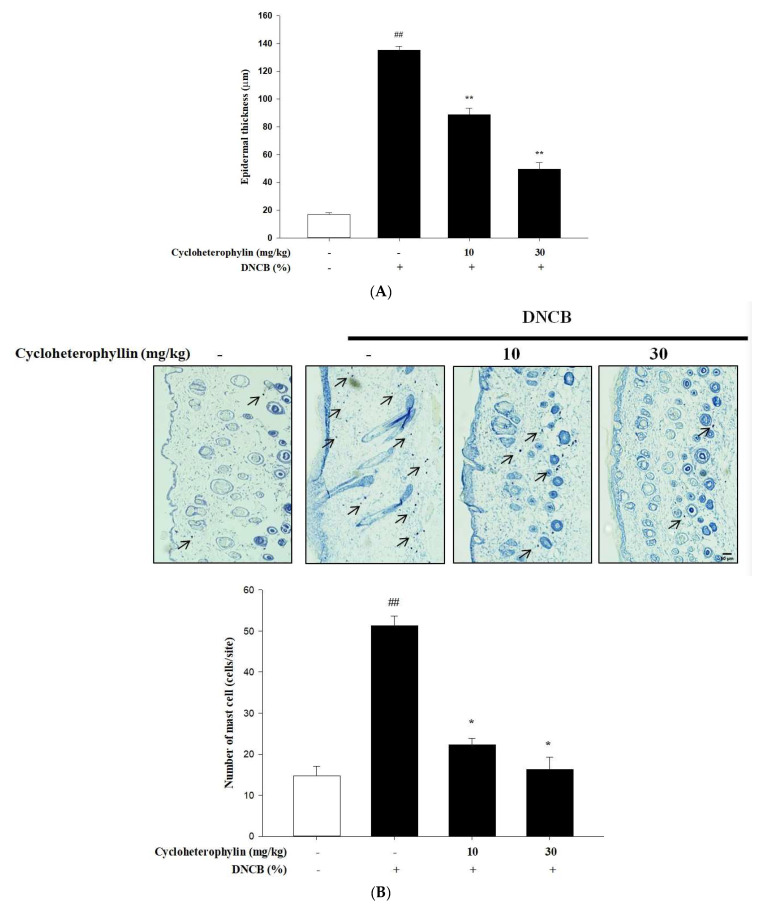
Effects of cycloheterophyllin on epidermal hyperplasia and mast cell infiltration. (**A**) Upper panel: histopathological variation due to DNCB induction was evaluated using hematoxylin–eosin staining (scale bar, 50 μm). Lower panel: quantitative analysis of epidermal thickness. (**B**) Toluidine blue staining; scale bar: 20 μm. Arrows indicate mast cells. Lower panel: number of mast cells. Values represent the mean ± SEM from at least three independent experiments. ^##^ *p* < 0.01 compared with the no-treatment condition; * *p* < 0.05 and ** *p* < 0.01 compared with the DNCB-induced group.

Finally, we used hematoxylin and eosin (H&E) staining and toluidine blue staining to detect epidermal hyperplasia and mast cell infiltration. As shown in Figure 8A, we can observe that DNCB does indeed cause a very serious epidermal hyperplasia. This hyperplasia is suppressed with the increase of the drug concentration (10 and 30 mg/kg). Further, we found that the infiltration of mast cells in the epidermis due to inflammation was also inhibited by the pretreatment of cycloheterophyllin (Figure 8B).

## 3. Discussion

The results of this study show that cyclohetrophyllin, a component isolated from tropical fruit trees, has not only anti-photoaging [8] and whitening effects [9], but also great potential in the future of inflammatory skin disease treatments. The experimental results showed that cyclohetrophyllin has not only a good antioxidant effect, but also an anti-inflammatory effect. Oxidation and inflammation are known to play very important roles in many diseases. Therefore, we infer that cycloheterophyllin also has very good development potential for other diseases caused by excessive oxidation and inflammation.

We found that cycloheterophyllin inhibited TNF-α/IFN-γ-induced IL-1β, IL-6, and IL8 expression in HaCaT. Epidermal keratinocytes are the main cellular component of the epidermis and may contribute to the pathogenesis of AD by producing pro-inflammatory genes [15]. Many studies have shown that keratinocytes produce TNF-α, IFN-γ, and IL-6, which are considered to be key to the inflammation medium [16,17]. Furthermore, the overproduction of cytokines by keratinocytes in AD skin lesions plays an important role in inflammation associated with atopic diseases [17,18]. Our findings show that cycloheterophyllin can effectively inhibit the expression of IL-6, IL-1β, and IL-8 in human keratinocytes, suggesting its therapeutic potential as an anti-AD agent. As for whether cycloheterophyllin has any effect on the expression of other cytokines and chemokines, this will be further studied in the future.

Various fruit trees have traditionally been used as folk medicines across civilizations [19]. The plant parts that are used in these traditional medicines include fruits, bark, leaves, stems, roots, branches, and sap [20]. They are widely used as a folk medicine for respiratory, digestive tract, and skin diseases [21]. In modern medicine, extracts from different parts of plants have been used for various therapeutic purposes. Most bioactive compounds found in plant extracts are prime candidates for their medicinal properties [22]. The phytochemicals of tropical fruit trees fall into three main groups: (1) phenolics, (2) carotenoids, and (3) terpenes and terpenoids [22]. The *Artocarpus* genus of the family Moraceae is a rich source of prenylated flavonoids and derivatives that have been studied phytochemically and biologically [23]. The fruit, root bark, and heartwood of *Artocarpus* *heterophyllus* have been isolated and found to contain many phenolic compounds with antioxidant and anti-inflammatory activities [23,24,25,26]. Previous phytochemical studies on *Artocarpus* *heterophyllus* have shown that flavonoids and 2-arylbenzofurans are present with cytotoxic, tyrosinase inhibitory, anti-inflammatory, and anti-respiratory burst activities [24,25,26,27,28,29]. Structurally, cycloheterophyllin is a flavone belonging to the flavonoid family and is a prenylflavone compound. In our study, cycloheterophyllin potently reduces the mRNA expression of proinflammatory cytokines caused by cytokines TNF-α and IFN-γ. A major consequence of MAPK phosphorylation is the activation of inflammatory transcription factors that act as downstream substrates of kinases to promote the secretion of inflammatory cytokines [11,13]. Previous studies have shown that phenolic compounds 11 and 30 from *Artocarpus heterophyllus* wood have anticancer potential through the MAPK pathway [25]. Therefore, the inhibition of proinflammatory cytokine expression by cycloheterophyllin may also reduce the expression of related cytokines through its effect on MAPK. As for which map kinase (ERK, JNK, or p38) contribution is relatively important, this needs to be investigated with related MAP kinase inhibitors in the future.

Our in vivo studies were performed in BALB/c mice with AD-like skin lesions treated topically with DNCB [11,14]. Histopathological analysis confirmed that cycloheterophyllin treatment reduced mast cell infiltration and DNCB-induced epidermal thickening, thereby alleviating DNCB-induced atopic skin symptoms in mice. It has been proposed that oxidative stress is involved in the pathogenesis of AD, which triggers skin inflammation by inducing epidermal keratinocytes to release pro-inflammatory cytokines and impair skin barrier function [30,31]. Therefore, antioxidants are believed to be beneficial in the prevention and/or treatment of AD. In our previous study, it was proven that cycloheterophyllin can resist the oxidative damage caused by hydrogen peroxide and UVA in skin fibroblasts. It can be understood that cycloheterophyllin has a very anti-oxidative effect in skin cells [8]. Our experimental results show that the ability of cycloheterophyllin to inhibit the release of cytokines and prevent epidermal water loss may also be closely related to the antioxidant properties of cycloheterophyllin. Mast cells play an important role in the pathogenesis of AD. Mast cells regulate inflammation and eosinophil activation by secreting multiple mediators [32]. Mast cell-derived histamine and other inflammatory mediators contribute to itch and inflammation in AD [33]. Further, our experimental results show that cycloheterophyllin can inhibit the scratching behavior caused by DNCB, and we also found that the infiltration of mast cells was significantly inhibited in the staining of tissue sections. Therefore, it is inferred that the effect on mast cells may also contribute to itching caused by anti-DNCB.

Topical administration has a faster effect on the administration site, but the dose is difficult to control. For AD animal studies, oral administration is more convenient and easier to control than topical administration [34,35]. Because AD symptoms are systemic and not limited to the skin, oral administration was chosen for this study. The results of our research show that cycloheterophyllin can achieve an anti-atopic dermatitis effect after oral administration. This shows that cycloheterophyllin can be developed not only for local administration but also for systemic treatment.

## 4. Materials and Methods

### 4.1. Materials

Cycloheterophyllin was isolated from the plant *Artocarpus heterophyllus Lam.* and dissolved in DMSO as previously described [26].

Sigma Chemical Co. (St Louis, MO, USA) was the source used to obtain 3-(4,5-Dimethylthiazol-2-yl)-2ami,5-diphenyltetrazolium bromide (MTT). Primary antibodies anti-p38, anti-ERK1/2, anti-JNK, anti-phospho-ERK1/2, anti-phospho-p38, and anti-phospho-JNK were purchased from Cell Signaling Technology (Beverly, MA, USA). The secondary antibodies were also purchased from Cell Signaling Technology. A Total RNA Isolation Kit (GeneDireX^®^, Vegas, NV, USA), an iScript™ cDNA Synthesis Kit (BIO-RAD, Hercules, CA, USA), and PowerUp™ SYBR™ Green Master Mix (Applied Biosystems™, Waltham, MA, USA) were used for Quantitative Polymer Chain Reaction (PCR) testing. A Pierce Protein Assay Kit (Pierce, Rockford, IL, USA) was used for a Western Blot Assay.

### 4.2. Methods

#### 4.2.1. Cell Culture and MTT Assay

Human immortalized keratinocytes (HaCaT cells) were a gift from Dr. Yih-Jing Lee of Fu Jen Catholic University. HaCaT cells were carefully cultured at 37 °C in DMEM containing 10% (*v/v*) FBS and 100 µg/mL antibiotics. Primary keratinocytes were isolated from human foreskin tissue and grown in Keratinocyte-SFM (Gibco BRL/Invitrogen, Carlsbad, CA, USA). In this study, primary keratinocytes were used between passages 2 and 4. Cell viability was assessed using the MTT assay. HaCaT cells (1 × 10^5^ cells/well) were seeded into 24-well plates and maintained at 37 °C in 5% CO_2_. After 24 h of culture, the cell culture medium was changed to a serum-free medium containing different concentrations of cycloheterophyllin for 1 h or 24 h. Then, MTT dye was added, and the dishes were incubated at 37 °C for an additional 3 h. The supernatant was then carefully removed, and the insoluble formazan crystals were dissolved in DMSO. Absorbance was measured at 540 nm using a spectrophotometer (Tecan Sunrise Basic Microplate Reader). DMEM, FBS, and antibiotics were purchased from Gibco-BRL (Grand Island, NY, USA).

#### 4.2.2. Quantitative Polymer Chain Reaction (PCR)

HaCaT cells were seeded in 3.5 cm dishes. Cells can reach 90% confluence after 24 h of quiescent growth. Cells were pretreated with cycloheterophyllin for 1 h and then stimulated with TNF-α/IFN-γ for 1 h, respectively. The cells were then scraped and centrifuged (16,000× *g*, 10 min, 4 °C) and the supernatant was removed. RNA was purified using the Total RNA Isolation Kit. According to the operation procedure of iScript™ cDNA Synthesis Kit, reagents were added one by one and operated under the specified conditions to convert RNA into cDNA. Additionally, PowerUp™ SYBR™ Green Master Mix was used. A total of 7.5 μL ddH_2_O, 2 μL cDNA, 0.25 μL forward and reverse primers, and 10 μL SYBR GREEN were added and mixed well. The primer sequences are shown in Table 1. RNA was then quantified using the ABI StepOnePlus™ Real-Time PCR System.

#### 4.2.3. Western Blot Assay

Western blots were used to analyze changes in various proteins in cells. HaCaT cells were seeded in 3.5 cm dishes. After cells reached 90% confluence and were starved for 24 h, they were pretreated with cycloheterophyllin for 1 h and then stimulated with TNF-α/IFN-γ for 1 h, respectively. After scraping, cells were crushed by sonication and centrifuged (13,200 rpm, 10 min, 4 °C). After centrifugation, the supernatant was taken, and protein was quantified using the Pierce Protein Assay Kit. Approximately 20–40 μg of protein was electrophoresed on a 10% SDS-polyacrylamide gel, followed by electroporation with PVDF membranes. After the transfer, the PVDF membrane was placed in a TBS-T solution (Tris-buffered salt/0.05% tween 20) containing 5% nonfat dry milk for 1 h with continuous shaking to avoid nonspecific binding. Then, the PVDF membrane was washed 3 times with TBS-T (30 min in total). After that, the primary antibody was added (diluted 1:1000). PVDF membranes were left overnight at 4 °C and then washed 3 times with TBS-T for 10 min each. Finally, after adding the secondary antibody for 1 h (diluted to 1:1000), the PVDF membrane was washed 3 times with TBS-T, then the developing solution was added, and the membrane was put into the chemiluminescence extraction system (BIOSTEP Celvin^®^) for photography.

#### 4.2.4. DNCB-Induced Atopic-Dermatitis-Like Skin Inflammation in Mice

Male BALB/c mice (8 weeks old) were purchased from the National Laboratory Animal Center of Taiwan. Mice were housed in a standard laboratory, and the temperature and humidity were controlled at 21 ± 2 °C and 50 ± 20%, respectively. Mice were housed in an animal center with filtered laminar airflow control and a 12-h light/dark cycle at Fu Jen Catholic University, New Taipei City, Taiwan. Mice were allowed to take water and food ad libitum. Experiments were performed with the approval of the Institutional Animal Care and Use Committee of Fu Jen Catholic University (Approval No. A10703).

**Figure 9:**
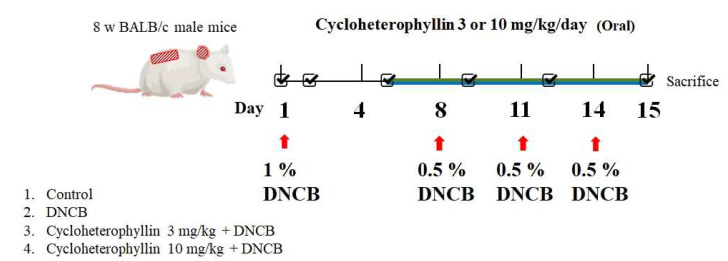
Experimental design of a mouse model of atopic dermatitis (AD)-like skin injury.

First, mice were divided into four groups: control group, DNCB group, and cycloheterophyllin (10 mg/kg and 30 mg/kg) plus DNCB group. In this in vivo experiment, cycloheterophyllin was dissolved in DMSO by ultrasonic shock, while DNCB was dissolved in 75% ethanol. The former was administered orally, while 100 μL and 20 μL of the latter were applied to the skin of the back and right ear, respectively. Three days before the experiment, mice were anesthetized, dorsal hair was removed, and a small measuring magnet (SCT-MAG-TF) was embedded in the back of each mouse’s hind foot. After standing for three days, it was confirmed that the mice were in good physical condition and had normal skin in the hair removal area on the back. Then the experiment was started. Relevant physiological values of mouse skin parameters, including TEWL, erythema, skin moisture, blood flow, ear thickness and number of scratches, were measured before the experiment. During the experiment, photographs were taken to document changes in the appearance of the skin and ears. Since the temperature and humidity in the environment could have had a great influence on the parameters to be measured on the skin surface, when evaluating the physiological parameters of the skin, the whole process was carried out in a room with constant temperature and humidity. The first stage (days 1–4) is the period of allergic atopic dermatitis. After measuring the basic physiological values of mice, 1% DNCB was evenly applied to the skin of the back and right ear. On the fifth day, oral cycloheterophyllin was started. The second stage (days 5 to 14) involved re-induction of atopic dermatitis. We evenly applied 0.5% DNCB to the skin of the back and right ear of mice in the three experimental groups. The next day, we tested and recorded the physiological values of the skin and took pictures. After completing all tests on day 15, the mice were euthanized with carbon dioxide (CO_2_), and the dorsal skin tissue and spleen were removed for subsequent experimental analysis (Figure 9).

Scratching behavior was tested by placing the animals in an observation cage (11 cm in diameter, MicroAct, Neuroscience, Tokyo, Japan). The scratching behavior of mice was automatically and objectively detected and assessed. Scratch behavior was measured for a total of 60 min. MicroAct uses the following analysis parameters to detect waves corresponding to continuous scratching behavior in mice: threshold, 0.05 V; interval between events, 0.05 s; minimum duration, 0.25 s; maximum frequency, 30 Hz; minimum frequency, 5 Hz.

#### 4.2.5. Statistical Analysis

Sigma-Plot software (version 10.0) was used for all statistical analyses of the data. All data are presented as mean ± SEM. Statistical significance was assessed by unpaired two-tailed Student’s *t*-test. Scientifically significant differences are indicated by *p*-values of less than 0.05 and 0.01. A single asterisk (*) indicates *p*-values of less than 0.05. Two asterisks (**) or two number signs (##) are indicated by *p*-values of less than 0.01 are indicated by.

## 5. Conclusions

From the above results and descriptions, we have demonstrated that cycloheterophyllin in *Artocarpus heterophyllus* has very good anti-inflammatory and antioxidant effects. These effects can contribute to the development of therapeutic drugs and combating AD.

## Figures and Tables

**Table 1:** Primer sequences for RT-qPCR.

Genes	Primers	Sequence (5′-3′)
IL-1β	Forward	CTC TCA CCT CTC CTA CTC ACT
	Reverse	ATC AGA ATG TGG GAG CGA AT
IL-6	Forward	ATC AGA ATG TGG GAG CGA AT
	Reverse	GGA CCG AAG GCG CTT GTG GAG
IL-8	Forward	ACT GAG AGT GAT TGA GAG TGG AC
	Reverse	AAC CCT CTG CAC CCA GTT TTC
GAPDH	Forward	CTG CTC CTG TTC GAC AGT
	Reverse	CCG TTG ACT CCG ACC TTC AC

## Data Availability

The data presented in this study is available in article.

## Sample Availability

Samples are not available from the authors.
